# Genetic specificity and potential for local adaptation between dengue viruses and mosquito vectors

**DOI:** 10.1186/1471-2148-9-160

**Published:** 2009-07-09

**Authors:** Louis Lambrechts, Christine Chevillon, Rebecca G Albright, Butsaya Thaisomboonsuk, Jason H Richardson, Richard G Jarman, Thomas W Scott

**Affiliations:** 1 Department of Entomology, University of California, One Shields Avenue, Davis, CA 95616, USA; 2 Génétique et Evolution des Maladies Infectieuses, UMR CNRS-IRD 2724, Centre de Recherche IRD, 911 Avenue Agropolis, B.P. 64501, 34394 Montpellier Cedex 5, France; 3 Department of Virology, Armed Forces Research Institute of Medical Sciences, 315/6 Rajvithi Road, Bangkok, 10400, Thailand; 4 Department of Entomology, Armed Forces Research Institute of Medical Sciences, 315/6 Rajvithi Road, Bangkok, 10400, Thailand

## Abstract

**Background:**

Several observations support the hypothesis that vector-driven selection plays an important role in shaping dengue virus (DENV) genetic diversity. Clustering of DENV genetic diversity at a particular location may reflect underlying genetic structure of vector populations, which combined with specific vector genotype × virus genotype (G × G) interactions may promote adaptation of viral lineages to local mosquito vector genotypes. Although spatial structure of vector polymorphism at neutral genetic loci is well-documented, existence of G × G interactions between mosquito and virus genotypes has not been formally demonstrated in natural populations. Here we measure G × G interactions in a system representative of a natural situation in Thailand by challenging three isofemale families from field-derived *Aedes aegypti *with three contemporaneous low-passage isolates of DENV-1.

**Results:**

Among indices of vector competence examined, the proportion of mosquitoes with a midgut infection, viral RNA concentration in the body, and quantity of virus disseminated to the head/legs (but not the proportion of infected mosquitoes with a disseminated infection) strongly depended on the specific combinations of isofemale families and viral isolates, demonstrating significant G × G interactions.

**Conclusion:**

Evidence for genetic specificity of interactions in our simple experimental design indicates that vector competence of *Ae. aegypti *for DENV is likely governed to a large extent by G × G interactions in genetically diverse, natural populations. This result challenges the general relevance of conclusions from laboratory systems that consist of a single combination of mosquito and DENV genotypes. Combined with earlier evidence for fine-scale genetic structure of natural *Ae. aegypti *populations, our finding indicates that the necessary conditions for local DENV adaptation to mosquito vectors are met.

## Background

Dengue viruses (DENV) are mosquito-borne viruses which, like many RNA viruses, exhibit substantial genetic diversity [1]. This diversity can be hierarchically organized in large clusters of lineages sometimes termed 'genotypes' (the terms lineage and genotype will be used interchangeably hereafter) within each one of the four distinct serotypes (DENV-1, -2, -3 and -4) [2]. In the last 200 years, the number of DENV lineages worldwide has been increasing exponentially [3], along with increasing epidemic frequency and occurrence of severe forms of the disease [4,5]. Dengue is now the most common human arthropod-borne viral (arboviral) disease and a major public health threat [6]. Although DENV genetic variation alone is not sufficient to completely explain the incidence of severe disease or the magnitude of outbreaks, there is compelling evidence for differences in virulence and epidemic potential among DENV lineages (reviewed in [2]). Understanding the evolutionary processes shaping the increasing diversity of DENV lineages will, therefore, provide important insights into the mechanisms regulating epidemics and pathogenicity associated with genetic variation among viruses [1].

The spatial distribution of DENV genetic diversity generally reveals a population structure whereby geographic subdivisions reflect greater gene flow within than between subdivisions (reviewed in [7]). Results from a recent study indicated that such a genetic structure can be observed on a fine spatial grid [8]; phylogenetic differences were detected among DENV isolates recovered from schools separated by only a few kilometers in rural Thailand. Although multiple DENV lineages co-circulated within individual schools, there was strong genetic differentiation among lineages between schools that remained stable over the 10-month sampling period [8]. Thus, despite frequent viral migration into the area, individual schools located a few kilometers apart represented distinct DENV evolutionary entities. A fundamental unanswered question concerns the relative influence of natural selection (adaptive evolution) and genetic drift (neutral evolution) on the genetic structure of DENV populations [9]. Although it is clear that stochastic processes play a significant role in shaping DENV genetic diversity [10,11], genetic signatures of adaptive evolution have been recurrently detected in natural DENV isolates [12-16].

Among evolutionary forces actively driving the evolution of arboviruses, vector-driven selection may play an important role by selecting lineages that are better suited for mosquito transmission [9]. For instance, the emergence of chikungunya virus (CHIKV) in the Indian Ocean in 2004, and subsequent spread to India and Europe was attributed to the acquisition of a single adaptive mutation that enhances transmission efficiency by *Aedes albopictus *[17,18]. This mutation confers a selective advantage in locations where *Ae. albopictus *predominates over *Ae. aegypti*, which is usually considered the primary vector of CHIKV. Similarly, the emergence of a new lineage of West Nile virus in North America was attributed to earlier and more efficient transmission by *Culex *mosquitoes relative to the lineage that initially colonized this part of the world [19]. DENV are no exception in this regard. Displacement of the American (AM) DENV-2 genotype by a Southeast Asian (SA) DENV-2 genotype in the Western Hemisphere was associated with more efficient infection and dissemination in *Ae. aegypti *of the SA than AM genotype [20-22]. Likewise, an invasive DENV-3 isolate from Sri Lanka infected and disseminated more efficiently in *Ae. aegypti *mosquitoes than a displaced, native DENV-3 isolate [23]. Interestingly, in both DENV examples above, outcompeted genotypes tended to cause mild disease whereas invasive genotypes correlated with more severe disease manifestations. This supports the view that enhanced vector transmission, among other factors, may contribute to the evolutionary success of lineages that are more virulent to humans [2].

The potential role of vector-driven selection in DENV evolution raises the question whether the genetic structure of DENV populations reflects, at least partly, that of their vectors. Indeed, *Ae. aegypti *distribution consists of a patchwork of genetically differentiated populations [24-27]. Because the vector competence of *Ae. aegypti *for DENV is in part genetically determined (reviewed in [28]), it has been hypothesized that the structure of DENV populations may partly result from the adaptation of viruses to the local vector genotypes [9]. Under this hypothesis, DENV transmission would be more efficient by local vector genotypes (sympatric vector-virus pairs) than by vector genotypes from distant populations (allopatric vector-virus pairs). Pathogen adaptation to local hosts has been reported in a variety of systems (reviewed in [29]), including malaria parasites and their mosquito vectors [30]. Evolution of local adaptation has been predicted when the pathogen has an evolutionary advantage over the host, such as higher mutation rate, shorter generation time, higher migration rate, and larger population size [31-33]. These conditions could undoubtedly apply to the DENV-*Ae. aegypti *system [9].

An additional necessary condition for the occurrence of DENV local adaptation to *Ae. aegypti *is that the genetic structure of mosquito populations must be coupled with some degree of genetic specificity of vector-virus compatibility. In other words, DENV transmission by *Ae. aegypti *must be determined, at least partly, by genotype × genotype (G × G) interactions. Such G × G interactions, whereby the infection outcome depends on the specific combination of host and pathogen genotypes, are found in many systems [34]. The effects of vector and virus genotypes, independently, on DENV transmission by *Ae. aegypti *are well-documented. Variation in vector competence for a reference DENV isolate among different geographic strains of *Ae. aegypti *has been repeatedly reported [35-38], and can often be directly correlated to genetic differences [39,40]. Reciprocally, DENV isolates have been shown to vary in their infectivity to a given mosquito strain [20,22]. Results from an earlier study showing some degree of interaction specificity between laboratory strains of mosquitoes and DENV serotypes and isolates [41] and from another study where SA genotypes of DENV-2 performed better than AM genotypes in two field-derived populations of *Ae. aegypti*, but not in a laboratory adapted colony [21] were suggestive of genetic specificity of vector-virus compatibility. However, the occurrence of G × G interactions between DENV and *Ae. aegypti *has not been formally quantified in natural populations.

Here, we measured the extent of G × G interactions between DENV and mosquito genotypes that are naturally interacting in the field. We challenged three *Ae. aegypti *isofemale families derived from a wild population sampled in 2007 in Ratchaburi, Thailand with three low-passage DENV-1 viruses that were isolated the same year from humans (in Bangkok, Kamphaeng Phet, and Ratchaburi, respectively). Following standard methods of quantitative genetics [34], we estimated the extent to which the outcome of the infection is determined by G × G interactions by measuring the statistical effect of isofemale family × virus isolate interactions on several indices of vector competence, defined as the intrinsic permissiveness of a vector to become infected and subsequently transmit a pathogen [42].

## Results

Overall, vector competence was scored in 333 female *Ae. aegypti*. Each combination of isofemale family and virus isolate was represented by 28–53 individuals (mean = 37, median = 36) divided into two experimental blocks of 11–28 individuals (mean = 18.5, median = 19). The three DENV-1 isolates used in this study were collected within a one-month period at three locations in Thailand (Bangkok: BKK, Kamphaeng Phet: KPP, and Ratchaburi: RTB) and had identical passage histories (Table 1). Estimated virus titers in the blood meals were remarkably similar across isolates and experimental blocks, with all titers ranging within a third of a log unit per ml (Table 1). This allowed us to assume that most of the phenotypic differences observed between isolates were due to genetic differences. Across families and isolates, 63.1% of females were infected, and 42.9% had a disseminated infection. Isofemale families differed significantly in wing length (S.S. = 0.052, *F*_2,57 _= 3.85, *P *= 0.027), indicating significant genetic variation in body size. The mean wing length (± SE) was 3.11 ± 0.018, 3.08 ± 0.018, and 3.04 ± 0.018 mm for families A, B, and C, respectively. Corresponding mean age at pupation (± SE) was 7.29 ± 0.035, 7.15 ± 0.037, and 7.28 ± 0.033 days, respectively. Although three data points are not enough to obtain a valid correlation, it is worth noting that the overall proportion of infected mosquitoes by family tended to be negatively correlated with their mean wing length (linear regression: R^2 ^= 0.97, *P *= 0.102), supporting the view that larger females are more resistant to dengue infection than small females [43].

**Table 1:** Description of DENV-1 isolates used in this study

**Isolate**	**Date collected**	**Location**	**Passage history**	**Blood meal titer, block 1 (FFUs/ml)**	**Blood meal titer, block 2 (FFUs/ml)**
BKK	27 July 2007	Bangkok	C6/36-5	5.5 × 10^6^	4.0 × 10^6^

RTB	24 July 2007	Ratchaburi	C6/36-5	5.0 × 10^6^	3.4 × 10^6^

KPP	19 Aug 2007	Kamphaeng Phet	C6/36-5	2.9 × 10^6^	2.0 × 10^6^

For each isolate, the date and location of collection, passage history, and blood meal titers for both experimental blocks are indicated. Blood meal titers were estimated by fluorescent focus assay (FFA) in C6/36 cells.

Two factors had a significant influence on end-point mortality of adult mosquitoes at 14 days post-infection (dpi). Mortality significantly differed between blocks (S.S. = 0.245, *F*_1,4 _= 19.5, *P *= 0.012), and marginally significantly between virus isolates (S.S. = 0.178, *F*_2,4 _= 7.08, *P *= 0.049). Although 5.8% of females in the first block died before 14 dpi, 16.3% of females in the second block did so, possibly due to the one-day age difference. Across blocks, end-point mortality was 5.3%, 11.1%, and 16.1% for the KPP, BKK, and RTB isolates, respectively. Mortality did not depend on the family-isolate combinations (S.S. = 0.131, *F*_4,4 _= 2.62, *P *= 0.19).

**Figure 1:**
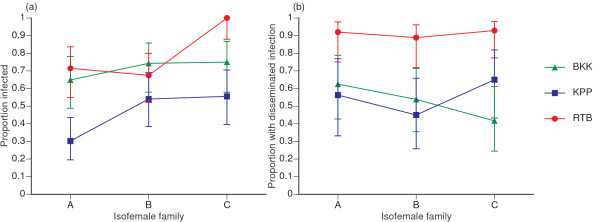
**Effect of family × isolate interactions on virus infection and dissemination**. (a) The proportion of mosquitoes with a midgut infection and (b) proportion of infected mosquitoes with a disseminated infection as a function of mosquito families and virus isolates. In both panels, three isofemale families from a Ratchaburi population (A, B, and C) are ranked on the x-axis according to the mean proportion of infected mosquitoes across isolates. Each line represents a single virus isolate (BKK: Bangkok; KPP: Kamphaeng Phet; RTB: Ratchaburi). Vertical bars show the confidence intervals of the proportions. Crossing lines give an indication of family × isolate interactions.

The proportion of infected mosquitoes strongly depended on the family, the isolate, and most importantly in the context of this study, their interaction (Table 2a; Figure 1a). The percentage of infected mosquitoes ranged from 30.2% to 100% among family-isolate combinations. The interaction effect appeared to be mainly due to the combination of the RTB isolate and mosquito family B for which the proportion of infected mosquitoes was lower than would have been expected from examination of the other combinations (Figure 1a). Because the family × isolate × block three-way interaction was not statistically significant, our results indicated that the effect of the family × isolate interaction was consistent across blocks (Table 2a). In other words, small differences in mosquito age or blood meal titer between blocks did not affect the probability of infection in a given mosquito-virus combination.

**Table 2:** Test statistics of categorical vector competence indices

		**(a) Proportion infected**		**(b) Proportion infected with disseminated infection**	
**Source of variation**	**d.f.**	**L-R χ^2^**	***P*-value**	**L-R χ^2^**	***P*-value**

Family	2	16.2	0.0003	2.34	0.3107

Isolate	2	29.4	<0.0001	38.8	<0.0001

Family*Isolate	4	14.8	0.0051	6.28	0.1793

Block	1	0	1.0	0	0.9882

Family*Block	2	0.64	0.7247	0.84	0.6574

Isolate*Block	2	0.64	0.7262	0.27	0.8753

Family*Isolate*Block	4	1.29	0.8634	11.9	0.0183

The table shows the nominal logistic regression for the proportion of (a) mosquitoes with detectable viral RNA in their bodies (thorax+abdomen) and (b) infected mosquitoes (excluding uninfected) with a disseminated infection in their head/legs (determined by FFA in Vero cells) as a function of mosquito isofemale families, virus isolates, experimental blocks, and their interactions.

In contrast, the proportion of mosquitoes with a disseminated infection among those successfully infected was only significantly influenced by the virus isolate (Table 1b). More than 90% mosquitoes infected by the RTB isolate had a disseminated infection whereas this percentage ranged from 40% to 70% for the BKK and KPP isolates (Figure 1b). Despite some degree of interaction indicated by crossing lines (Figure 1b), the family × isolate interaction was not statistically significant, possibly due to variation between experimental blocks as indicated by the significant family × isolate × block three-way interaction (Table 1b). Moreover, exclusion of the 123 uninfected mosquitoes reduced statistical power of this analysis.

**Figure 2:**
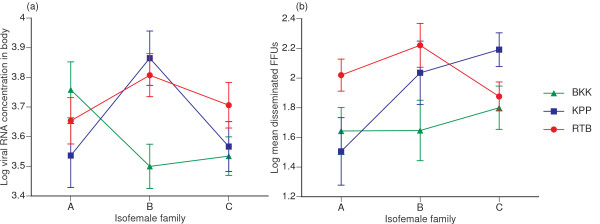
**Effect of family × isolate interactions on viral RNA concentration in mosquito bodies and virus titer in heads/legs**. (a) The log-transformed viral RNA copy number per μl of homogenized body (thorax+abdomen) in infected mosquitoes and (b) log-transformed mean number of fluorescent focus units (FFUs) in the head/legs of mosquitoes with a disseminated infection as a function of mosquito families and virus isolates. In both panels, three isofemale families from a Ratchaburi population (A, B, and C) are ranked on the x-axis according to the mean proportion of infected mosquitoes across isolates (consistently with Figure 1). Each line represents a single virus isolate (BKK: Bangkok; KPP: Kamphaeng Phet; RTB: Ratchaburi). Each point represents the mean and vertical bars are the standard errors of the means. Crossing lines give an indication of family × isolate interactions.

Viral RNA concentrations in the body of infected mosquitoes and the number of fluorescent focus units (FFUs) in the head/legs of mosquitoes with a disseminated infection varied substantially among family-isolate combinations, with varying ranking orders of viral isolates for each isofemale family (Figure 2). Both variables were significantly influenced by the family × isolate interaction, with no significant difference between experimental blocks (Table 3). Regardless of their ranking order in a given mosquito family, the general profile of each isolate shared some degree of similarity between viral RNA concentration and numbers of disseminated FFUs (Figure 2), possibly reflecting differing viral growth capacities in different mosquito genetic backgrounds. Viral RNA concentration in infected mosquitoes did not appear to be correlated to the proportion of infected mosquitoes or the proportion of mosquitoes with a disseminated infection, indicating that the probability of these events may be largely independent of the efficiency of viral genome replication.

**Table 3:** Test statistics of continuous vector competence indices

	**(a) Viral RNA concentration in body**				**(b) Mean virus titer in head/legs**			
**Source of variation**	**d.f.**	**S.S.**	***F***	***P*-value**	**d.f.**	**S.S.**	***F***	***P*-value**

Family	2	0.63	2.01	0.1374	2	1.53	2.22	0.1127

Isolate	2	0.59	1.88	0.1550	2	1.45	2.10	0.1266

Family*Isolate	4	2.09	3.33	0.0115	4	3.58	2.60	0.0394

Block	1	0.02	0.14	0.7126	1	0.83	2.42	0.1223

Family*Block	2	0.01	0.03	0.9668	2	0.44	0.63	0.5328

Isolate*Block	2	0.29	0.93	0.3949	2	0.83	1.20	0.3053

Family*Isolate*Block	4	0.87	1.38	0.2417	4	1.27	0.92	0.4530

Error	192	30.1			125	43.1		

Results from analysis of variance of (a) viral RNA concentration in the bodies (thorax+abdomen) of infected mosquitoes and (b) mean virus titer (determined by FFA in Vero cells) in the head/legs of mosquitoes with a disseminated infection as a function of mosquito isofemale families, virus isolates, experimental blocks, and their interactions.

## Discussion

Results of our experiment strongly support the hypothesis that vector competence of *Ae. aegypti *for DENV-1 is governed by G × G interactions. With the exception of virus dissemination success, all of the indices of vector competence we examined (midgut infection success, viral RNA concentration in bodies, virus titer in head/legs) were at least partly determined by the specific combination of mosquito family and virus isolate. The use of a randomized complete block design allowed us to rule out the possibility of environmental bias by confirming the consistency of family × isolate interactions effect across experimental blocks.

Our conclusions are based on the premise that family × isolate interactions reliably approximate G × G interactions [34]; i.e., in our design observed phenotypic differences truly reflect the underlying genetic differences between mosquitoes and viruses. The use of isofemale families to assess the genetic basis of a trait assumes that the ratio of among- to within-families variations is proportionate to the heritable variation for that trait [44]. This assumption is reasonable as long as environmental variation in rearing conditions or parental effects do not differ between families. We ensured homogeneity by maintaining the F_0_-F_2 _parental generations under standard laboratory conditions and rearing the F_3 _individuals under strictly identical conditions. Non-genetic differences in virus infectivity were minimized by growing the viruses simultaneously under the same conditions and exposing mosquitoes to very similar blood meal titers (Table 1). Uncontrolled, slight differences in blood meal titers did not appear to influence results. For instance, the RTB isolate produced the highest average values across families for all vector competence indices, although the BKK isolate had the highest blood meal titers in both experimental blocks. Although virus amplification in cell culture may have imposed a selective pressure on the original viruses, the low number of passages allowed us to assume that we still had isolates representative of wild-type viral genetic diversity. Overall, it seemed reasonable to conclude that our approach provided a reliable estimate of G × G interactions in this system.

It is remarkable that we were able to detect G × G interactions using a very restricted experimental design (three mosquito families × three virus isolates). Observing a highly significant effect of the interaction term with limited statistical power indicates that G × G interactions likely represent a strong determinant of vector competence in natural *Ae. aegypti *populations, which display a much higher level of genetic polymorphism than was captured by our three isofemale families sampled at a single location. We expect G × G interactions to be even more prominent when mosquito genotypes from more genetically distinct populations are examined. Indeed, a nested analysis of *Ae. aegypti *mitochondrial haplotype frequencies in Thailand showed that while 57% of the total variation was found within collections, 18% and 25% of that variation were found among samples collected within a distance of 25 km and more than 100 km apart, respectively [25]. Moreover, because the three DENV-1 isolates used in this study were all collected in Thailand in 2007, it is likely that they are closely genetically related. We expect that G × G interactions will occur to a greater extent when the analysis includes more distantly related viral lineages or even different serotypes.

A practical implication of our results is that the conclusions drawn from one particular vector-virus combination (such as one mosquito population challenged with a reference DENV isolate) are not likely to be representative of other combinations. This challenges the broad-scale relevance of genetic loci associated with vector competence that were identified in laboratory-tractable systems. A recent meta-analysis showed that quantitative trait loci (QTL) controlling host resistance against one pathogen isolate were on average recovered in only 24% of the cases where infections with different isolates were investigated; i.e., each particular host-pathogen combination was based on a different set of QTL and epistatic interactions [45]. G × G interactions may help to explain why the QTL controlling midgut infection by a Jamaican DENV-2 isolate in highly selected lines of *Ae. aegypti *[46,47] did not correlate with those previously identified in field-derived *Ae. aegypti *challenged with a Puerto Rican DENV-2 isolate [48]. Likewise, it sheds a new light on a study where DENV-2 isolates of the SA genotype performed better than isolates of the AM genotype in two field-derived *Ae. aegypti *populations, but not in a laboratory-adapted colony [21].

Although additional studies are warranted to confirm the impact of G × G interactions on actual virus transmission through an infectious bite, their occurrence during DENV incubation in *Ae. aegypti *provides important insights into the mechanisms shaping DENV population structure and evolution. It helps to explain why despite strong genetic bottlenecks during mosquito transmission, major DENV variants are generally conserved in both vertebrate and arthropod hosts [49,50]. Indeed, G × G interactions indicate that elimination of viruses that are incompatible with local mosquito genotypes may counteract the effect of genetic drift in the form of purifying selection at the population level. Likewise, the observed spatial clustering of DENV lineages on a restricted spatial scale [8] may in fact be promoted by location-specific vector-driven selection. Over time, genetic specificity of vector-virus compatibility would combine with the genetic structure of *Ae. aegypti *populations to drive the adaptation of DENV to increased compatibility with local mosquito genotypes [9]. Interestingly, it is worth noting that in our experiment, while the three isofemale vector families were derived from samples collected in Ratchaburi, all of the vector competence indices were highest, on average, for the viral isolate from that same location (RTB). Although this does not constitute firm evidence for local adaptation of DENV, it is consistent with the hypothesis that DENV transmission may be achieved with greater success by sympatric than by allopatric vector-virus pairs. Inclusion of mosquito families from different populations in a similar design will help to more definitively address this question. Future research using multiple replicates of sympatric and allopatric pairs will determine the extent of local adaptation in this system. It is clear that factors other than vector-mediated population structure contribute to the evolution of certain DENV lineages; i.e., 'cosmopolitan' genotypes that are found across broad geographical distributions. We speculate, however, that to some extent the spatial organization of DENV populations may reflect the geographical distribution of *Ae. aegypti *genotypes, which would be similar to what was demonstrated (at the species level) for Mexican populations of *Plasmodium vivax *and their *Anopheles *vectors [30].

## Conclusion

We demonstrate that the vector competence of *Ae. aegypti *for DENV-1 is determined by G × G interactions in which potential for mosquito infection and virus transmission depends on the specific combination of vector and virus genotypes. This observation challenges the general relevance of genetic loci controlling vector competence that were identified using a single combination of mosquito and virus genotypes. The combination of G × G interactions and spatial genetic structure of vector populations is consistent with the potential for DENV adaptation to local vectors. Mosquito vector-driven selection may play a more important role in DENV microevolution than previously thought.

## Methods

### General design

Because we wanted to (i) use a representative sample of a natural mosquito population and (ii) minimize parental effects due to the variability in environmental conditions [51], we used F_3 _isofemale families of *Ae. aegypti *generated from field-sampled immatures (larvae and pupae) raised for two generations in the laboratory. Isofemale families consist of the progeny of individual females. They are classically used in studies of quantitative genetics to investigate the genetic basis of a trait (e.g., [52]). Although *Ae. aegypti *females can mate multiple times, they are thought to be monandrous due to male accessory gland proteins that are transferred during copulation and induce subsequent sexual refractoriness [53]. Based on the assumption that sibs are genetically more similar than non-sibs, one can estimate the extent to which a trait has a genetic basis by comparing the phenotypic variations observed within and between families [44]. Using this approach genetic effects are confounded with potential maternal effects, which can be controlled by rearing the parental generations under standard homogenous conditions. Our experiment was based on a reciprocal cross-infection design involving three *Ae. aegypti *isofemale families and three DENV-1 isolates. The experiment was duplicated in two separate experimental blocks by splitting F_3 _mosquitoes from the same batch into two groups and infecting each group on two successive days with the same viral culture harvested one day apart. This procedure allowed us to assess the repeatability of results while controlling for nuisance factors introduced by duplication of the experiment (e.g., mosquito age, virus titer). We measured G × G interactions with the statistical effect of the family × isolate two-way interaction on vector competence [34]. We estimated the repeatability of the results with the statistical significance of the family × isolate × block three-way interaction as an indication of the degree of consistency of the two-way interaction effect across blocks.

### Mosquitoes

The F_0 _generation was initiated with a large number (>1,500) of immatures collected from multiple (3–6) artificial containers in each of 10–12 households in Don Thako, Muang district, Ratchaburi (Thailand) during September 2007. Adults were allowed to emerge in the laboratory, mate randomly, and feed on anesthetized hamsters as a blood source. Eggs were collected and stored on dry pieces of paper towel maintained under high humidity. Mosquitoes in F_1_-F_3 _generations were raised under standard insectary conditions at 27 ± 1°C, high humidity, and under 12:12 hour light:dark cycle. Eggs were hatched synchronously by placing them under low pressure for 30 min and larvae were reared in 25 × 40 cm plastic trays filled with 1.5 l of deionized water at a density of approximately 200 individuals per tray. F_1_-F_2 _larvae were fed on a 1:1 mix of ground puppy chow and bovine liver powder (0.05 g/tray on days 0, 1, and 2; 0.1 g/tray on day 3; 0.2 g/tray on day 4; 0.3 g/tray on day 5; and 0.2/tray on days 6 and 7). Adults were housed in large cages with permanent access to 10% sucrose. F_1 _females were fed on defibrinated sheep blood (Quad Five, Ryegate, MT) through pieces of desalted porcine intestine stretched over water-jacketed glass feeders. F_2 _females were allowed to mate randomly for three days and then housed individually. They were daily offered a blood meal on a human arm and allowed to lay eggs on wet filter papers. In order to obtain a large number of F_3 _females in each family, egg batches from multiple (4–6) gonotrophic cycles were combined and hatched simultaneously. F_3 _larvae were reared at a low density (approximately 125 per tray) to maximize their survival. Larvae of each family were reared in several trays that were moved daily at random between the shelves of the insectary to minimize any uncontrolled environmental variation. They were fed on a 1:1 mix of ground puppy chow and bovine liver powder (0.02 g/tray on day 0; 0.04 g/tray on day 1; 0.06 g/tray on day 2; 0.08 g/tray on day 3; 0.1 g/tray on day 4; 0.2 g/tray on day 5; and 0.1/tray on days 6 and 7). Female body size was estimated for each F_3 _isofemale family by measuring the wing lengths of a random sample of 20 individuals per family. Wings were measured from the tip (excluding the fringe) to the distal end of the alula with a precision of ± 0.01 mm using a dissecting microscope equipped with a micrometer. All experimental infections were conducted using F_3 _females.

### Infection

We used three DENV-1 isolates that were recovered during July-August 2007 as part of routine surveillance diagnostic procedures at the Armed Forces Research Institute of Medical Sciences (AFRIMS) Bangkok laboratory from the serum of clinically ill dengue patients attending hospitals in Bangkok, Ratchaburi, and Kamphaeng Phet. Each diagnostic isolate underwent a strictly identical passage history (Table 1). Five passages in cell culture was the minimum number required to obtain a titer sufficiently high to infect mosquitoes orally using an artificial blood meal. Confluent cultures of *Ae. albopictus *cells (C6/36, ATCC #CRL-1660) in 6-well plates were inoculated with virus at a multiplicity of infection of 0.01 and incubated at 28°C under 5% CO_2_. Half of the supernatant was replaced with fresh medium 7 dpi. Cells and medium were harvested at 11 and 12 dpi to prepare the infectious blood meal of experimental blocks 1 and 2, respectively. The blood meal consisted of 1:1 mix of defibrinated sheep blood (Quad Five) and virus suspension. Three- to six-day-old (block 1) and four- to seven-day-old adult mosquitoes (block 2) deprived of sucrose for 24 hours were offered an infectious blood meal for 30 min via membrane feeding as described above. Samples of the blood meal were saved for subsequent viral titration by fluorescent focus assay (FFA) in C6/36 cells [54]. Preliminary trials showed that there was no detectable decrease in blood meal titer over a period of 30 min. After feeding, mosquitoes were briefly knocked-down with CO_2 _and fully engorged females were transferred to paper cups and held in a Precision incubator at 27 ± 1°C and under 12:12 hour light:dark cycle and supplied with 10% sucrose *ad libitum*. High humidity in the incubator was maintained with containers filled with water.

### Vector competence

The intrinsic ability of mosquitoes to transmit DENV was assessed 14 dpi with two standard phenotypes used in vector competence studies: (i) midgut infection and (ii) viral dissemination from the midgut to other tissues. Midgut infection was determined by detecting the presence of virus in mosquito bodies (thorax and abdomen). Viral dissemination was determined by detection of virus in mosquito legs and heads. Virus titers in the head and the legs of the same individual were strongly correlated among infected mosquitoes (linear regression: R^2 ^= 0.522, *P *< 0.0001). We, therefore, used their average in the quantitative analyses of virus dissemination. We used both categorical (infection and dissemination status) and continuous measures (viral RNA concentration in bodies, virus titer in heads/legs) of vector competence components. We also recorded the proportion of dead mosquitoes at 14 dpi to account for any differential mortality between experimental groups. Mosquitoes were anesthetized with triethylamine (Sigma-Aldrich, St. Louis, MO), and their legs and heads were removed individually and transferred separately into 0.5 ml of mosquito diluent (MD), consisting of 20% heat-inactivated fetal bovine serum (FBS) in Dulbecco's phosphate-buffered saline (PBS) with 50 μg/ml penicillin/streptomycin, 50 μg/ml gentamicin, and 2.5 μg/ml fungizone. The remainder of the mosquito bodies were placed individually into 0.7 ml of MD, and all samples were stored at -80°C before processing. Samples were thawed on ice and homogenized in a mixer mill (Qiagen, Valencia, CA) at 24 cycles/sec for 2 min. Infected bodies were screened by TaqMan^® ^quantitative RT-PCR (qRT-PCR). Although viral RNA molecules are not equivalent to infectious virions, our conservative qRT-PCR detection threshold (10^4 ^viral RNA copies per sample, see below) allowed us to consider that positive bodies contained >1 infectious virion because viral RNA copy numbers are 100- to 10,000-fold higher than virus titers [21,55]. Disseminated infections in the heads and legs of mosquitoes whose body was positive by qRT-PCR was determined by FFA in green monkey kidney cells (Vero cells, ATCC #CCL-81) as described in [54]. Mosquitoes whose bodies were negative by qRT-PCR were considered uninfected and their legs and heads were not processed further. We verified the validity of the screening scheme by confirming that the bodies, legs, and heads of a random sub-sample of individuals negative by qRT-PCR were also negative by FFA.

### Quantitative RT-PCR

Viral RNA was quantified in mosquito bodies by a serotype-specific, one-step TaqMan^® ^qRT-PCR method modified from [56]. Briefly, RNA was extracted and purified using a semi-automated Prism 6100 Nucleic Acid Prepstation (Applied Biosystems, Foster City, CA). A sample of 300 μl of each mosquito homogenate was pre-filtered and lysed with 300 μl of 2× Nucleic Acid Purification Solution, and 500 μl of the resulting lysate was deposited in a Purification Tray. Isolated RNA was washed once with 500 μl of Wash Buffer 1, once with 500 μl of Wash Buffer 2, twice with 300 μl of Wash Buffer 2, and finally eluted in 60 μl of Elution Solution. qRT-PCR was carried out in 96-well plates with a 7900HT Fast Real-Time PCR system (Applied Biosystems) using a TaqMan^® ^One-Step RT-PCR Master Mix Reagents kit (Applied Biosystems). Each sample was assayed in a 50 μl reaction containing 10 μl of RNA template, 0.5 μM of forward primer, 1 μM of reverse primer, and 0.25 μM of fluorogenic DENV-1 specific probe. Forward and reverse primer sequences NS5F and NS5R reported in [56] were used to generate an amplicon of 104 bp within the *NS5 *gene. The dual-labeled DENV-1 specific probe sequence used in this study (5'-[6-FAM]-CTCAGAGACATATCAAAGATTCCAGC-[BHQ1]-3') was slightly modified from the DSQ1 sequence reported in [56] so that the target region of the *NS5 *gene was strictly identical among our three isolates (as confirmed by sequencing). The thermal profile consisted of an RT step at 48°C for 30 min, 10 min of *Taq *polymerase activation at 95°C, followed by 40 cycles of PCR with 30 sec of denaturation at 95°C and 1 min of annealing/extension at 60°C. RNA solutions of known concentration were synthesized by *in vitro *transcription (IVT) and used as RNA standards for absolute quantification across plates [56]. A standard RT-PCR was carried out on viral genomic RNA using a forward primer including a T7 promoter sequence. The T7 forward primer sequence was the IVT NS5F sequence reported in [56] and the reverse primer sequence was the cFD4 sequence reported in [57]. PCR products were cleaned up using the QIAquick PCR Purification kit (Qiagen) and 1 μg of DNA was subjected to IVT using the T7 MAXIscript IVT kit (Ambion, Austin, TX) to generate a transcript of 480 bp containing the target *NS5 *region. IVT products were treated with TurboDNase (Ambion) at 37°C for 15 min and RNA was cleaned using the RNeasy MinElute kit (Qiagen). RNA was re-suspended in DEPC-treated water and molecular concentration was calculated by converting the optical density at 260 nm into the molecular copy number [56]. Solutions of concentrations ranging from 10^7 ^to 10^1 ^RNA copies/μl were aliquoted and stored at -80°C until used to generate a standard curve for each TaqMan^® ^plate. Baseline fluorescence was set automatically to account for differing starting RNA quantities between samples. The threshold level of fluorescence for threshold cycle (Ct) determination was optimized manually so that the slope of the standard curve was as close as possible to the theoretical value -3.32 which corresponds to 100% PCR efficiency. Positive and negative controls were included on each plate. For all plates the standard curve had an R^2 ^> 0.985 and the detection limit was 10^3 ^RNA copies/μl; i.e., 10^4 ^RNA copies per 10 μl of RNA template.

### Fluorescent focus assay

Virus titers were quantified using a tissue-culture assay based on immuno-fluorescent detection of infectious foci developing in cell monolayers [54]. Blood meal titers were assayed in C6/36 cells to provide a relevant estimate of viral infectivity to mosquito cells, whereas virus titers in mosquito heads and legs were assayed in Vero cells because we were interested in the eventual infectivity to a mammalian host. Cells were grown in 75-cm^2 ^flasks in Dulbecco's Modified Eagle Medium with 4,500 mg/l of D-glucose and L-glutamine (DMEM, Invitrogen) supplemented with 10% FBS, 1.5 g/l sodium bicarbonate, 100 U/ml penicillin, and 100 μg/ml streptomycin. Eight-well chambered slides were seeded with Vero or C6/36 cells at a density of 2.5 × 10^5 ^or 5.0 × 10^6 ^cells/well, respectively, and incubated for 24 hours at 37°C (Vero) or 28°C (C6/36) under 5% CO_2_, to produce a confluent monolayer. Ten-fold serial dilutions of blood meal samples or head/legs homogenates were inoculated onto cell monolayers in a final volume of 50 μl/well. Viral adsorption was allowed to proceed for one hour at 37°C (Vero) or 28°C (C6/36) under 5% CO_2_, rocking the slides every 15 min. At the conclusion of the adsorption, an overlay of growth medium with 5% FBS, and 0.8% carboxymethylcellulose (CMC, Sigma-Aldrich) was added in a final volume of 0.5 ml/well. DENV infectious foci develop faster in Vero than in C6/36 cells [54]. After an incubation of 48 hours at 37°C (Vero) or 72 hours at 28°C (C6/36) under 5% CO_2_, the overlay medium was removed from the infected monolayers and the cells were washed twice with cold PBS. Infected monolayers were fixed for 10 min in ice-cold absolute methanol (Sigma-Aldrich) and washed once with PBS. Slides were incubated for one hour with a mouse anti-dengue complex primary antibody clone D3-2H2-9-21 (Millipore, Temecula, CA) diluted 1:200 in PBS containing 0.2% bovine serum albumin (PBS-BSA 0.2%). After three washes in PBS-BSA 0.2%, slides were incubated for 30 min with a goat anti-mouse fluorescein-conjugated secondary antibody (Millipore) diluted 1:50 in PBS-BSA 0.2%, followed by three washes in PBS-BSA 0.2% and a final wash in distilled water. Cells were mounted in Vectashield anti-fading medium (Vector Laboratories, Burlingame, CA) and observed under a 20× objective on an Olympus Provis fluorescence microscope equipped with a FITC filter. The total number of fluorescent foci in each well was visually counted and virus titers were calculated as fluorescent focus units (FFUs) per ml. Reading was done blindly by number-coding and randomizing samples on the slides.

### Data analysis

We first analyzed differences in body size between isofemale families by comparing wing lengths using a one-way analysis of variance (ANOVA). Then, because our experiment fulfilled the characteristics of a randomized complete block design [58], we used full-factorial analyses including the factors mosquito family, virus isolate, experimental block, and all their interactions. Categorical variables (infection and dissemination status) were analyzed with multi-way logistic nominal regressions, whereas continuous variables (viral RNA concentration in bodies, virus titer in heads/legs, mortality) were analyzed with multi-way ANOVAs. In order to satisfy the assumptions of the statistical tests (in particular, normality of the residuals), we used a logarithmic transformation of viral RNA concentrations and virus titers and an arcsine-transformation of mortalities. The proportion of mosquitoes with a disseminated infection and concentration of viral RNA in bodies were analyzed among infected mosquitoes (i.e., excluding uninfected mosquitoes). The average virus titer of heads and legs was analyzed among mosquitoes with a disseminated infection (i.e., excluding mosquitoes that did not have a disseminated infection). Differences were considered statistically significant at *P *< 0.05. All statistical analyses were performed with the software JMP version 5.1.2 .

## Authors' contributions

LL conceived the study, performed the research, analyzed the data, and wrote the manuscript. CC helped to design the study and draft the manuscript. RGA participated in the vector competence assays. BT carried out the isolation and amplification of the viruses. JHR coordinated the field collection of mosquitoes. RGJ coordinated the collection of virus isolates. TWS participated in the design and coordination of the study, and helped to draft the manuscript. All authors read and approved the final manuscript. The opinions or assertions contained herein are the private views of the authors and are not to be construed as reflecting the official views of the United States Army, Royal Thai Army, or the United States Department of Defense.
